# Anthocyanin metabolic engineering of *Euphorbia pulcherrima*: advances and perspectives

**DOI:** 10.3389/fpls.2023.1176701

**Published:** 2023-05-15

**Authors:** Edmundo Lozoya-Gloria, Fernando Cuéllar-González, Neftalí Ochoa-Alejo

**Affiliations:** Departamento de Ingeniería Genética, Unidad Irapuato, Centro de Investigación y de Estudios Avanzados del Instituto Politécnico Nacional, Irapuato, Gto., Mexico

**Keywords:** *Euphorbia pulcherrima*, poinsettia, Christmas star, anthocyanins, metabolic engineering

## Abstract

The range of floral colors is determined by the type of plant pigment accumulated by the plant. Anthocyanins are the most common flavonoid pigments in angiosperms; they provide a wide range of visible colors from red-magenta to blue-purple, products of cyanidin and delphinidin biosynthesis, respectively. For the floriculture industry, floral color is one of the most important ornamental characteristics for the development of new commercial varieties; however, most plant species are restricted to a certain color spectrum, limited by their own genetics. In fact, many ornamental crops lack bluish varieties due to the lack of activity of essential biosynthetic enzymes for the accumulation of delphinidin. An example is the poinsettia (*Euphorbia pulcherrima* Willd. ex Klotzsch), the ornamental plant symbol of Christmas and native to Mexico. Its popularity is the result of the variety of colors displayed by its bracts, a kind of modified leaves that accumulate reddish pigments based mainly on cyanidin and, to a lesser extent, on pelargonidin. The commercial success of this plant lies in the development of new varieties and, although consumers like the typical red color, they are also looking for poinsettias with new and innovative colors. Previous research has demonstrated the possibility of manipulating flower color through metabolic engineering of the anthocyanin biosynthesis pathway and plant tissue culture in different ornamental plant species. For example, transgenic cultivars of flowers such as roses, carnations or chrysanthemums owe their attractive bluish colors to a high and exclusive accumulation of delphinidin. Here, we discuss the possibilities of genetic engineering of the anthocyanin biosynthetic pathway in *E. pulcherrima* through the introduction of one or more foreign delphinidin biosynthetic genes under the transcriptional control of a pathway-specific promoter, and the genome editing possibilities as an alternative tool to modify the color of the bracts. In addition, some other approaches such as the appropriate selection of the cultivars that presented the most suitable intracellular conditions to accumulate delphinidin, as well as the incorporation of genes encoding anthocyanin-modifying enzymes or transcription factors to favor the bluish pigmentation of the flowers are also revised.

## 1 Introduction

**Figure 1:**
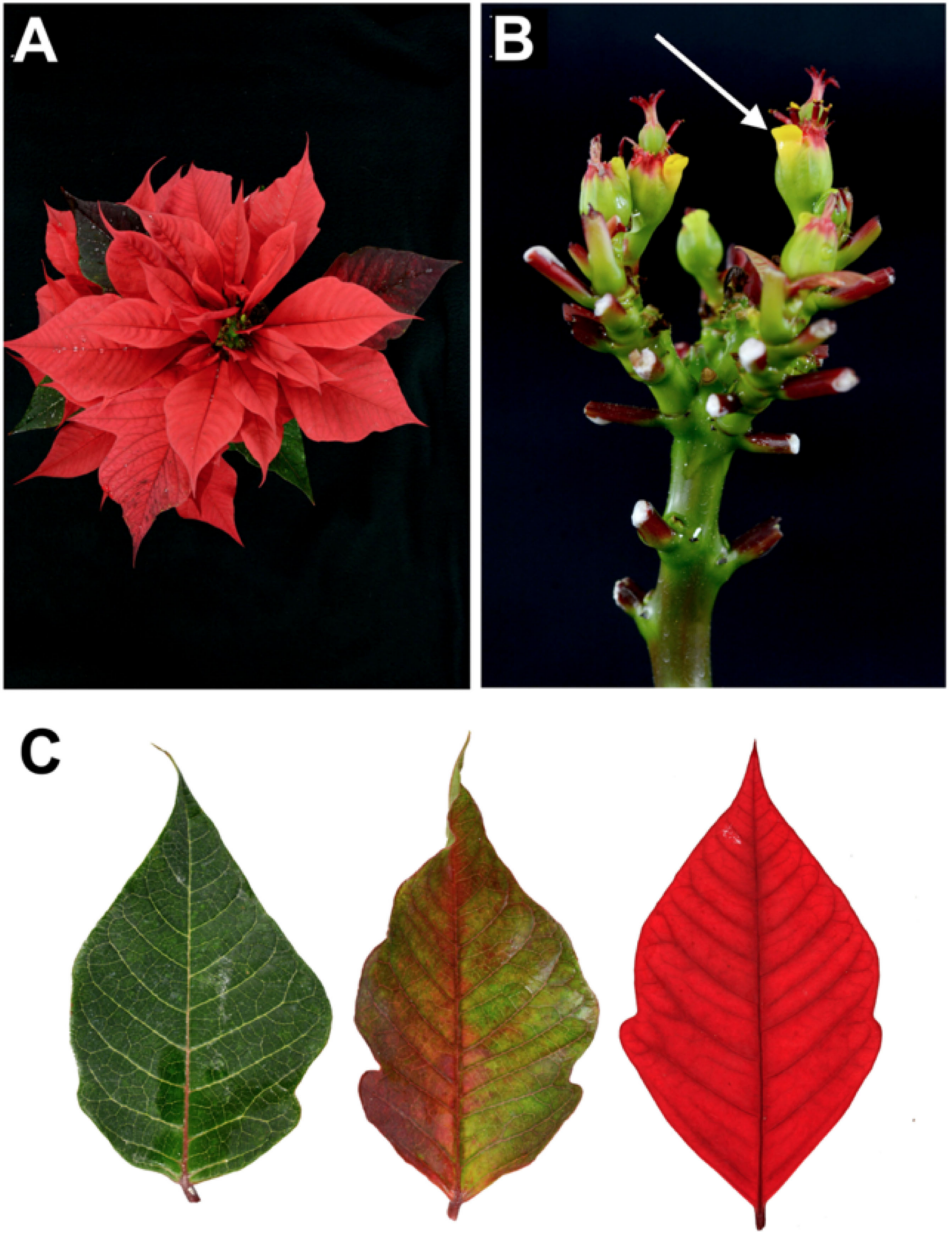
*Euphorbia pulcherrima* Willd. ex Klotzsch. **(A)** Inflorescence of the commercial poinsettia cultivar Prestige Red. **(B)** Cyathia forming the inflorescence, each cyathium composed of a yellow nectary on the edge (arrow) and a pistil in the center surrounded by stamens. **(C)** Gradual pigmentation changing process from a green leaf (left), a partially pigmented red bract (middle), and a completely red bract (right).

Plants produce flowers with different forms, sizes, colors, and aromas; these organs are the ones specialized in sexual reproduction and the perpetuation of the species through the fertilization of the ovules by the pollen to form seeds with the intervention of diverse pollinators. Therefore, flowers attract insects, birds, and some other animals by producing diverse pigments, aromas, and nectars. Pigments of different chemical nature are biosynthesized and accumulated in the petals of flowers; among them, anthocyanins are the most common, abundant, and widely distributed in plants. Flowers are highly appreciated as ornamental issues because of their enormous diversity of forms, sizes, colors, and aromas. All these characteristics can be modified or improved through traditional breeding techniques; however, this process usually takes years or even decades. One of the traits that can be manipulated by modern biotechnological techniques is the flower color to generate novel cultivars, which are not found in nature; this has been possible by the metabolic engineering of anthocyanin biosynthesis in different ornamental plant species such as *Rosa* spp., *Chrysanthemum* spp., and *Dyanthus* spp. to produce plants bearing blue flowers (Fukui et al., 2003; Katsumoto et al., 2007; Tanaka and Brugliera, 2013; Noda et al., 2013; Noda et al., 2017). *Euphorbia pulcherrima* Willd. ex Klotzsch (poinsettia) is an ornamental plant that typically synthesizes reddish cyanidin anthocyanins in modified leaves known as bracts, which form a kind of “false” flowers (**Figure 1**). Cultivars with variations in the color of bracts (white, orange, or pink, among others) are also commercialized as attractive products. Therefore, there is a commercial opportunity for the application of biotechnological approaches to generate poinsettias with unnatural colors in the bracts, such as blue ones. For this aim, it is necessary to have *in vitro* plant regeneration and transformation protocols to manipulate the anthocyanin biosynthesis pathway in *E*. *pulcherrima*; fortunately, different authors have reported on these issues (Geier et al., 1992; Clarke et al., 2008; Islam et al., 2013; Danial and Ibrahim, 2016);, and even on the possibility of genome editing (Nitarska et al., 2021). Some possibilities and difficulties or limitations to accomplish this goal are discussed here.

## 2 From the family Euphorbiaceae to the genus *Euphorbia*

Euphorbiaceae is the sixth family with the greatest diversity of plants; it includes approximately 8,700 species comprised in 320 genera. Its distribution is sub-cosmopolitan, predominantly with species in tropical and subtropical regions, although they are also distributed in temperate zones of both hemispheres. Various forms of growth are described: from tall forest trees to lianas, shrubs, annual and perennial herbs, geophyte, and floating aquatic succulents (Dressler, 1961; Webster, 1994; Steinmann, 2002).

The Euphorbiaceae family is important because several of its members are cultivated for food, medicine, production of poisons, oils and fats, waxes, gums, rubber, components for paints, varnishes, and as ornamentals. It is estimated that in Mexico this family is represented by 43 genera and 782 species, of which the largest genera are: *Euphorbia* (241 spp., 31%), *Croton* (124 spp., 16%), *Acalypha* (108 spp., 14%), *Jatropha* (48 spp., 6%) and *Phyllanthus* (41 spp., 5%) (Pascual and Correal, 1992; Steinmann, 2002).

*Euphorbia* genus is morphologically the most diverse of flowering plants with 2,160 species registered (Horvath et al., 2011), which can be shrubs, trees, succulent plants, or herbs provided with latex; with alternate or opposite leaves, usually membranous, entire, or toothed. The flowers grow in a cup-shaped structure known as a cyathium, an inflorescence made up of several frequently bare flowers, included in an involucre inside of which both the male flowers (in the periphery) and the female (in the center) are located. This is represented by a sessile ovary at the apex of the pedicel; the fruit is a trilocular capsule; and the stems and other organs often tend to present reddish coloration (Sánchez, 1980; Rzedowski and Rzedowski, 2005; Horvath et al., 2011). The main use of some species included in this genus is ornamental, highlighting poinsettia (*Euphorbia pulcherrima* Willd. ex Klotzsch) (Rzedowski and Rzedowski, 2005) also known with other names as Christmas flower, Christmas star, lobster plant, painted leaf, Mexican flame leaf, Easter flower (Flor de Pascua), and Nochebuena (Christmas Eve).

## 3 Botanical characteristics and history

*Euphorbia pulcherrima* Willd. ex Klotzsch is a native species to Mexico, with its center of origin north of Guerrero and Morelos states (Trejo et al., 2012). Poinsettia is a perennial shrubby plant that, in the wild form, can reach 5 m height. It is distributed in deciduous tropical forest regions, found along the Pacific coast from Sinaloa state, Mexico, to Guatemala (Steinmann, 2002; Trejo et al., 2012). It produces latex, a white and milky liquid excreted when tissues are wounded or cut; the flowers, devoid of sepals and petals, contain a single female flower surrounded by male flowers; on the edge, there is a yellow appendage called nectary whose function is to attract pollinators, all enclosed in the cyathium (**Figure 1**) (Ecke et al., 1990; Hartley, 1992; Shanks, 2004; Taylor et al., 2011).

Poinsettia is also characterized by responding to photoperiods, and flowering begins as the nights become longer than days (Recasens Pahí and Flores Llurba, 1983; Hartley, 1992). Under these conditions, the vegetative apex ends the growth of the stem, then the development of the cyathia begins (the first forms only staminate flowers), the last two internodes do not elongate, and the three upper leaves become bracts; the axillary buds of the three upper leaves grow, although they immediately form another cyathium subtended by a bract, and the process is repeated, resulting in a cluster with the floral arrangement of three main branches. The showy and colorful portions, characteristic of this plant, are modified leaves called bracts (**Figure 1**) (Recasens Pahí and Flores Llurba, 1983; Hartley, 1992; Shanks, 2004).

In Mexico, before Spanish invasion, poinsettia was cultivated by the Aztecs in an area near Taxco in Guerrero state; they called it “cuetlaxochitl”, which in Nahuatl means “withering flower”. The radiant flowers were worshiped as a symbol of purity by natives and kings, and were used to make purple dyes and make-up, whereas latex was used for medicinal purposes. Because of the typical flower displayed by poinsettias during the Christmas season, in the 17th century Franciscan priests used them at the Feast of the Holy Manger, a commemorative procession of the Nativity (Ecke et al., 1990; Fry, 1994; Lee, 2000; Taylor et al., 2011). This is the first record of poinsettias used for the Christmas season.

Poinsettias were first introduced to the United States of America (USA) in 1825 by Joel Robert Poinsett, a botanist and the first American ambassador to Mexico from whom “poinsettia” name was acquired. Poinsett collected and shipped samples of plant material to Greenville County in South Carolina, where they were subsequently distributed to various botanical gardens. In 1829 Robert Buist, a horticulturist from Philadelphia in Pennsylvania sold the first poinsettia plant (Hartley, 1992; Fry, 1994; Lee, 2000; Lack, 2011; Taylor et al., 2011).

## 4 Development of varieties and economic value

After its introduction to Philadelphia and in the late 19th century, poinsettia was propagated and marketed by horticulturists as an outdoor plant and for warmer climates, even as early as the 1900s it was sold as a cut flower. In 1923, a new era in sales began when a horticulturist named Paul Ecke proposed that the flower would be perfect for Christmas (Hartley, 1992; Fry, 1994; Taylor et al., 2011). Years later, in the mid-1950s, poinsettia production began in the US with the introduction of a series of changes for its cultivation, such as: 1) the selection, reproduction and development of more attractive varieties; shortening of the internodes distance in the inflorescence, making them more compact and with greater ramification, with rounded contours and more colorful bracts; 2) the production went from soil to a potted greenhouse, to standardize the plantation and; 3) the promotion so that the plant became an essential element in the December holidays (Fry, 1994; Lee, 2000; Vázquez and Castañeda, 2004; Trejo et al., 2018).

Over nearly 200 years of cultivation outside of Mexico, poinsettias have been transformed from those tall, unbranched wild ancestors to the dense, leafy, and brightly colored profile of domesticated ones (Trejo et al., 2018). Likewise, and through marketing efforts, poinsettia has become the symbol plant of Christmas, of cultural and economic importance, recognized as one of the most substantial potted ornamental plants, cultivated and marketed in large quantities throughout the five continents (Hartley, 1992; Fry, 1994; Lee, 2000; Lack, 2011; Trejo et al., 2018).

The highest production of poinsettias is in the United States; for 2016 it was estimated a sale value of 140 million dollars (USDA, 2021). In Mexico for 2021, a production of 17.3 million plants was registered in 257 hectares, with a profit of 668 million pesos (SIAP, 2021). The six main producing entities are: Morelos, with 6.9 million plants; Mexico City with 3.6 million; Puebla with 3.1 million; Jalisco with 1,7 million; the State of Mexico with 953 thousand and Michoacan with 765 thousand. In addition, it generates more than three thousand direct jobs and close to nine thousand indirect ones (SAGARPA, 2018).

Currently, there are around 300 varieties of commercial poinsettias, most of them developed in the US and to a lesser extent in some countries of the European Union (Hartley, 1992; Fry, 1994; Trejo et al., 2018). In Mexico, some efforts have been made to generate some poinsettia cultivars and hybrids (Vargas-Araujo et al., 2017; Canul-Ku et al., 2018; García Pérez et al., 2019). On the market, they are available in various sizes, shapes, response times for flower induction, exhibiting bract colors such as red, yellow, pink, white, striped, and marbled (Cabrera et al., 2006); being the color the most important ornamental characteristic to consider in the development of new poinsettia varieties (Taylor et al., 2011) and, although consumers like the traditional red color, they also look for cultivars with different styles and new colors (López et al., 2010).

## 5 Colors of poinsettia, opportunity area for plant biotechnology

In poinsettia, light signals captured by the photoreceptors induce the flowering process, the production of chlorophyll decreases, and the biosynthesis and gradual accumulation of anthocyanins begins (**Figure 1**) (Pomar and Barceló, 2007; Meng and Liu, 2015). Anthocyanins are in the upper and lower epidermis and in the adjacent mesophyll cells (palisade and spongy) (Moustaka et al., 2020). The types of anthocyanin present in poinsettia are those derived from cyanidin and pelargonidin, predominantly more of the former, regardless the variety and red range it exhibits (Asen, 1958; Slatnar et al., 2013; Gu et al., 2018).

It is important to mention that, to date, no anthocyanins derived from delphinidin have been identified in poinsettia; therefore, the lack of bluish varieties in this plant is probably due to the lack of this pigment. This agrees with reports of roses, carnations, chrysanthemums, dahlias, clematis, or lilies, where the lack of delphinidin-based anthocyanins is due to the absence of an active gene encoding the flavonoid 3’, 5’-hydroxylase enzymes (F3’5’H) (Fukui et al., 2003; Katsumoto et al., 2007; Brugliera et al., 2013). On the other hand, despite efforts to obtain blue cultivars through conventional breeding techniques, the genetic and physiological barriers of these flowers prevent the development of blue varieties (Nishihara and Nakatsuka, 2010). Therefore, there is a lot of interest to apply metabolic engineering techniques to ornamental plants because these offer the possibility to manipulate the type of resulting pigments (Tanaka and Brugliera, 2006; Sasaki and Nakayama, 2015).

## 6 Plant pigments

There are more than 300,000 species of flowering plants, classified into more than 400 families; in nature, they exhibit various forms, fragrances, and colors. Floral organs are usually showy and colorful, a characteristic that is attributed to the type of accumulated pigment (Nishihara and Nakatsuka, 2010). Plant pigment is generated by its own electronic structure, which interacts with sunlight to alter the wavelengths, which are then reflected by plant tissue, then colors results from a combination of residual wavelengths, and the specific perceived color will depend on the observer. Humans detect wavelengths between 380 and 730 nm, with the visible spectrum ranging from violet, indigo, blue, green, yellow, orange and red (Davies and Schwinn, 2004; Schwinn and Davies, 2004; Zhao and Tao, 2015).

In plants, pigments are found in fruits, seeds, leaves, and flowers; and fulfill various functions, primarily chlorophyll with its role in photosynthesis; although there are also other pigments that are structural components of the photosynthetic apparatus and protect it from photooxidative damage. Pigments also participate in plant-light interactions, particularly in response to ultraviolet radiation, and help to defend plants against pathogens. However, its main function is to provide color to the flowers and fruits, to attract pollinators and seed dispersal agents (Forkmann, 1991; Davies and Schwinn, 2004; Nishihara and Nakatsuka, 2010) to perpetuate the species. Plant pigments have been used extensively by humans, from their use in textiles, cosmetics, henna tattoos, and natural food coloring, to their important nutraceutical role for human health. In agriculture, the contribution of plant pigments is important for the consumer’s choice of fruits, vegetables, and floriculture products (Davies and Schwinn, 2004). For the ornamental industry, color is an important determinant of product quality that not only affects ornamental merit, but directly influences its commercial value (Mol et al., 1999; Schwinn and Davies, 2004; Zhao and Tao, 2015).

It is known that the color of flowers is regulated by physical factors such as temperature, which affects the accumulation of pigments by reducing or increasing them, resulting in lighter or darker colors at high and low temperatures, respectively. Intensity and quality of pigments are influenced by light, and water controls chromaticity through its effect on the accumulation and distribution in the vacuole. Among chemical factors, there are the soil pH where plant develops; phytohormones particularly growth retardants and mineral nutrients. However, the main determinant of flower color is of course the type of plant pigment it contains (Davies and Schwinn, 2004; Zhao and Tao, 2015).

There are many kinds of plant pigments with long and complex structures. They are identified and divided based on a common chemical structure and on their biosynthesis, having four main groups: 1) chlorophylls, present in all photosynthetic plants giving their green color; 2) carotenoids, liposoluble terpenoid pigments ubiquitously distributed in the plant kingdom, are essential components of photosystems, and commonly divided into carotenes and xanthophylls and located in plastids of leaves, roots, seeds, fruits and flowers. They produce yellow colors as e.g., *Helianthus annuus* (sunflower), red colors e.g., *Solanum lycopersicum* (tomato), pink colors e.g., flowers of *Tulipa* genus and orange. Combined with flavonoids, they also result in purple colors e.g., *Cymbidium* (orchid) and black e.g., *Viola* (pansy); 3) betalains, nitrogenous pigments derived from tyrosine, water soluble and stored in vacuoles, commonly divided into betacyanins and betaxanthins and are the most restricted group found in few families of the order Caryophyllales. These can produce an intense purple color e.g., *Bougainvillea* (bougainvillea) and *Portulaca* (purslane) genera; and 4) flavonoids, these are widely diverse and taxonomically extended compounds in angiosperms, gymnosperms, ferns, and bryophytes. Although they provide only few colors, they are the most common pigment group in flowers and the most studied (Davies and Schwinn, 2004; To and Wang, 2006; Tanaka et al., 2008; Tanaka et al., 2010; Chandler and Brugliera, 2011; Zhao and Tao, 2015).

## 7 From flavonoids to anthocyanins

Flavonoids are a group of secondary metabolites belonging to the phenylpropanoid class, synthesized, and glycosylated in the cytosol and then transported into the vacuoles (Tanaka et al., 2008). They are in almost all plant tissues and fulfill various biological functions such as signaling during nodulation, protection against pathogens, absorbing UV-B rays protecting plants from possible damage; additionally, they have antioxidant capacity and play an important role in the response mechanism to biotic and abiotic stresses; moreover, they influence auxin transport and participate in pollen fertility. However, its best-known function is to provide color to the flowers to attract pollinators, and seed and fruit dispersers. Flavonoids are the most important group of pigments and provide the widest range of visible colors to plants (Forkmann, 1991; Davies and Schwinn, 2004; Schwinn and Davies, 2004; Chandler and Tanaka, 2007; Falcone Ferreyra et al., 2012; Zhao and Tao, 2015).

**Figure 2:**
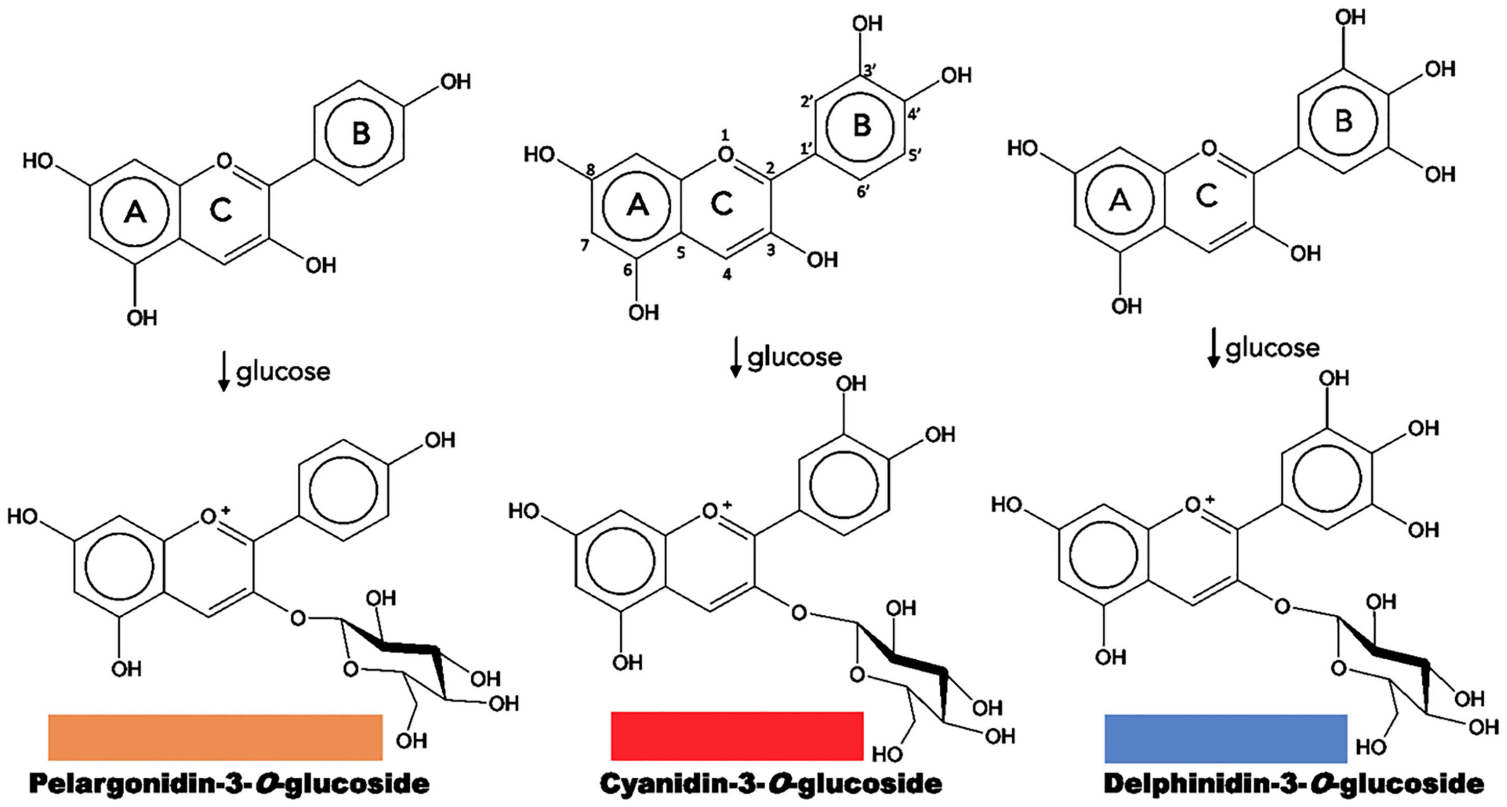
Structure of natural anthocyanidins and anthocyanins. Flavonoid structure is depicted on the upper central part. The color of each anthocyanin type is illustrated.

Chemically, flavonoids are a collection of water-soluble substances, which have a 15-carbon (C15) base structure with a C6-C3-C6 arrangement, consisting of two phenyl rings (A and B rings) connected by a bridge of three carbons that usually form a third ring (C ring) (**Figure 2**). There are about 7,000 different types of flavonoids, mainly determined by the oxidation degree of the C ring. However, only some of them can absorb light in the visible spectrum region (400 - 800 nm) and can be considered as pigments. Within the flavonoids, anthocyanins are the most predominant, while chalcones, aurones, flavones and flavonols have a more limited role (Schwinn and Davies, 2004; To and Wang, 2006).

Anthocyanins are specific flavonoid structures named anthocyanidin joined to a sugar moiety, or anthocyanidin glycosides; sugar groups may be glucose, rhamnose, xylose, galactose, and arabinose. They are soluble in water, methanol, ethanol, and acetone; and exhibit a characteristic absorption peak at 500 – 550 nm in the visible region. They are the most abundant flavonoid pigments (630 different types) and widespread in angiosperms, which are synthesized in the cytosol and stored in the vacuole; and they can be found in other organs such as leaves, seeds, fruits, and pollen. Anthocyanins produce colors ranging from pink to black, and the diversity of colors results from the level of anthocyanidin’s oxygenation, and the number of substituents added to these chromophores (Davies and Schwinn, 2004; Schwinn and Davies, 2004; Chandler and Tanaka, 2007; Tanaka et al., 2008).

Most anthocyanins derive from three common types: pelargonidin, with colors from orange to red; cyanidin, with magenta derivatives; and delphinidin, resulting in a range of purples and blues (**Figure 2**). The differences result from the number of hydroxyl groups in B ring, a higher hydroxylation has a bluish effect on the color. On the other hand, many plants tend to accumulate more than one type of anthocyanin or even a mixture with other flavonoids such as flavones and flavonols, creating combinations that provide a greater color variation (Schwinn and Davies, 2004; Chandler and Tanaka, 2007; Tanaka et al., 2008; Tanaka et al., 2010; Zhao and Tao, 2015).

**Figure 3:**
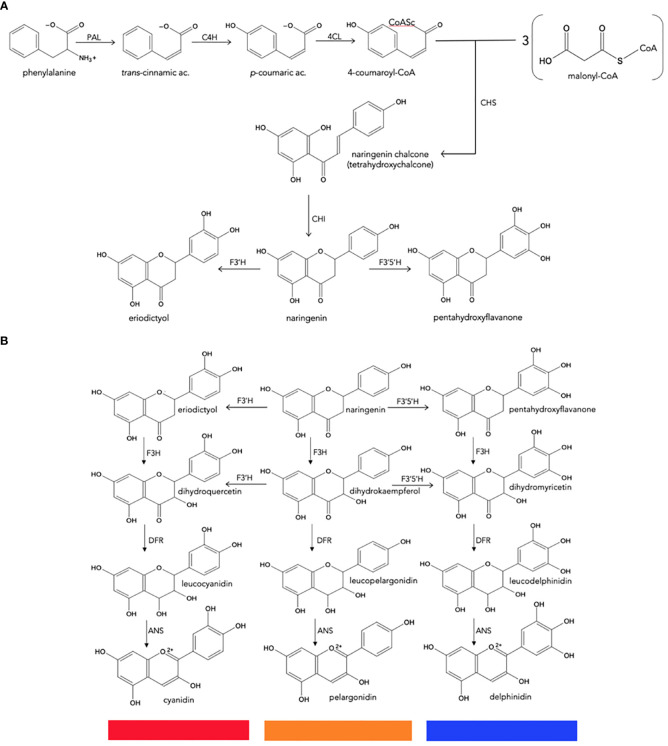
Biosynthesis of natural anthocyanidins. **(A)**, General metabolism of phenylpropanoids to produce naringenin chalcone. **(B)**, Conversion of naringenin to final anthocyanidins. CHS, chalcone synthase; CHI, chalcone isomerase; F3H, flavanone 3-hydroxylase; F3′H, flavonoid 3′-hydroxylase; F3′5′H, flavonoid 3′,5′-hydroxylase; DFR, dihydroflavonol 4-reductase; ANS, anthocyanidin synthase.

Because of their importance as secondary metabolites, the biosynthetic pathways of most plant pigments are well defined at genetic and enzymatic levels, and their regulation at the transcriptional level is also known (**Figure 3**) (Davies and Schwinn, 2004; Tanaka et al., 2005; Chandler and Tanaka, 2007; Zhao and Tao, 2015).

## 8 Anthocyanin biosynthesis in plants

Anthocyanin biosynthesis is highly conserved at the structural and functional levels in plants (Tanaka et al., 2008; Petroni and Tonelli, 2011). It can be divided into three stages (**Figure 3**): the first involves the conversion of phenylalanine to 4-coumaroyl-coenzyme A (CoA), which is the key substrate that feeds the flavonoid pathway. This conversion is catalyzed by the phenylalanine ammonia lyase (PAL), cinnamate-4-hydroxylase (C4H) and 4-coumaroyl: CoA ligase (4CL) enzymes. The second stage is the formation of dihydroflavonols through a series of conversions beginning with chalcone synthase (CHS), enzyme that catalyzes the condensation of one molecule of 4-coumaroyl-CoA and three of malonyl-CoA to synthesize the first precursor of all flavonoids, a chalcone molecule usually naringenin-chalcone (2’,4,4’,6’-tetrahydroxychalcone [THC]), which is unstable; then the chalcone isomerase (CHI) enzyme catalyzes the isomerization of chalcone, a reaction that stabilizes C ring to form naringenin-flavanone, a product also required for the synthesis of flavones and flavonols. Hydroxylation has a key impact on anthocyanin color (Chandler and Tanaka, 2007), and the most important variation is the B ring hydroxylation patterns. An increase in hydroxylation causes a significant change in color from the extreme magenta to the blue end of the spectrum. This increase is due to the specific activity of the F3’H and F3’5’H hydroxylases, both members of the Cyt P450 family. They use molecular oxygen, NADPH as cofactors and the dihydroflavonol dihydrokaempferol (DHK) as a substrate to catalyze hydroxylation at carbon 3- or carbons 3- and 5- of the B ring, to produce dihydroquercetin (DHQ) (cyanidin precursor) and dihydromyricetin (DHM) (delphinidin precursor), respectively (**Figure 2**). Its activity determines the type of anthocyanin and, consequently, the color of the flower (Schwinn and Davies, 2004; Seitz et al., 2006; Seitz et al., 2007).

Flavanone-3-hydroxylase (F3H) enzyme from the 2-oxoglutarate dehydrogenase (OGDH) family is involved in the formation of dihydroflavonols through the hydroxylation of naringenin-flavanone and other flavanones at C3; and then the flavonoid 3’-hydroxylase (F3’H) and flavonoid 3’,5’-hydroxylase (F3’5’H), which are key reactions in the metabolism of flavonoids. The third stage is the formation of various anthocyanidins from dihydroflavonols, which is catalyzed by the dihydroflavonol 4-reductase (DFR) enzyme that has an important role because determines the type of anthocyanins formed and the color of the flower; it belongs to the short-chain dehydrogenases/reductases family and is encoded by one or more genes. This enzyme can catalyze a NADPH-dependent reduction reaction for three types of dihydroflavonols: dihydromyricetin (DHM), dihydroquercetin (DHQ), and dihydrokaempferol (DHK) to produce their corresponding colorless leucoanthocyanidins; the anthocyanidin synthase (ANS) enzyme, which acts at the last stage of the pathway, catalyzes the conversion of leucoanthocyanidin to the colorful anthocyanins. The synthesized anthocyanidins are then modified through a series of glycosylations and methylations catalyzed by UPD-glucose: flavonoid-3-glucosyl transferase (UFGT) and methyltransferase (MT), to form stable anthocyanins (Tanaka et al., 1998; Schwinn and Davies, 2004; Tanaka et al., 2005; Zhao and Tao, 2015). Some biosynthetic enzymes come from families of enzymes, such as 2-oxoglutarate dehydrogenase (OGDH), cytochrome P450 (Cyp 450), and glycosyltransferases (GTs), suggesting that plants recruit these enzymes from pre-existing metabolic pathways (Tanaka et al., 2008).

The great diversity of anthocyanins is because they can form more complex structures through modifications such as glycosylations, acylations and methylations. Another important characteristic is that the final color of the flower is determined by different factors that contribute to the intensity and spectrum of the color; for example, the pH of the vacuole affects the intensity, tone or even loss of color. In an acid medium, orange to red pigments are stable, while in slightly acid to neutral solutions purple to violet appear, and blue is only stable in alkaline solutions. Another phenomenon is co-pigmentation, usually with flavones and flavonols that, in addition to stabilizing anthocyanin, have a bluish effect, increasing the intensity of the color. In some species, anthocyanins interact with metal ions such as iron, aluminum, and magnesium to form stable blue pigment structures. An intrinsic factor is the shape of the cells where the pigment is stored, which influences light refraction and reflection (Goto and Kondo, 1991; Schwinn and Davies, 2004; Tanaka et al., 2005; Falcone Ferreyra et al., 2012; Zhao and Tao, 2015). In addition to these factors, the synthesis of anthocyanins is under strict spatial and temporal control involving the development of the plant, the environment, and the season (Davies and Schwinn, 2004; Schwinn and Davies, 2004), as is the case of poinsettia.

Transcriptional regulators that activate structural anthocyanin biosynthesis-related genes in different plant species include transcription factor members of protein families containing R2R3-MYB domains, bHLH (single helix-loop-helix) and conserved WD40 repeats; these participate as a single ternary complex of MYB-bHLH (MYC)-WD40 (MBW) transcription factors (Petroni and Tonelli, 2011; Falcone Ferreyra et al., 2012).

## 9 Anthocyanins in *E. pulcherrima*

In poinsettia, the red color is provided by the bracts, modified leaves whose axillary buds have differentiated to form the inflorescence, in response to photoperiod. Poinsettia plants exposed to short days (11 h of light and 13 h of darkness), for a period of eight weeks, develop the cyathium, and the bracts complete their red pigmentation. This color is due to the gradual accumulation of anthocyanins and the decrease in chlorophyll production during floral induction (Kannangara and Hansson, 1998; Pomar and Barceló, 2007). Light signals captured through photoreceptors can activate various physiological processes to induce metabolic responses such as biosynthesis and accumulation of anthocyanins in plant tissues (Meng and Liu, 2015).

The type of pigment synthesized in poinsettia has been studied by Lawrence et al. (1939), who pointed out the anthocyanins as the main component. Subsequently, Asen (1958), characterized these anthocyanins in three cultivars exhibiting different red color intensities. The same types of cyanidins and pelargonidins were found, predominantly more of the former and regardless of the variety. In the work of Slatnar et al. (2013), they identified eleven different types of anthocyanins; however, six of them in very low concentrations, one of which was delphinidin-3-(2G-xylosyl-rutinoside). The five anthocyanins identified in a red poinsettia cv. Mira Red, in high concentrations were: cyanidin-3-galactoside, cyanidin-3-glucoside, cyanidin-3-rutinoside, pelargonidin-3-glucoside and pelargonidin-3-rutinoside. Its quantification by high performance liquid chromatography coupled to mass spectrometry (HPLC/MS), yielded average values of 1081, 2211, 1228, 1485 and 655 mg kg^-1^ fresh weight, respectively. Interestingly, in the pink variety cv. Mars Pink, the same anthocyanins were found but, in less quantity (approximately three times less than the red one), and curiously, the white cv. Mars White contained the same, although in very low concentrations (approximately 150 times less) (Slatnar et al., 2013).

**Figure 4:**
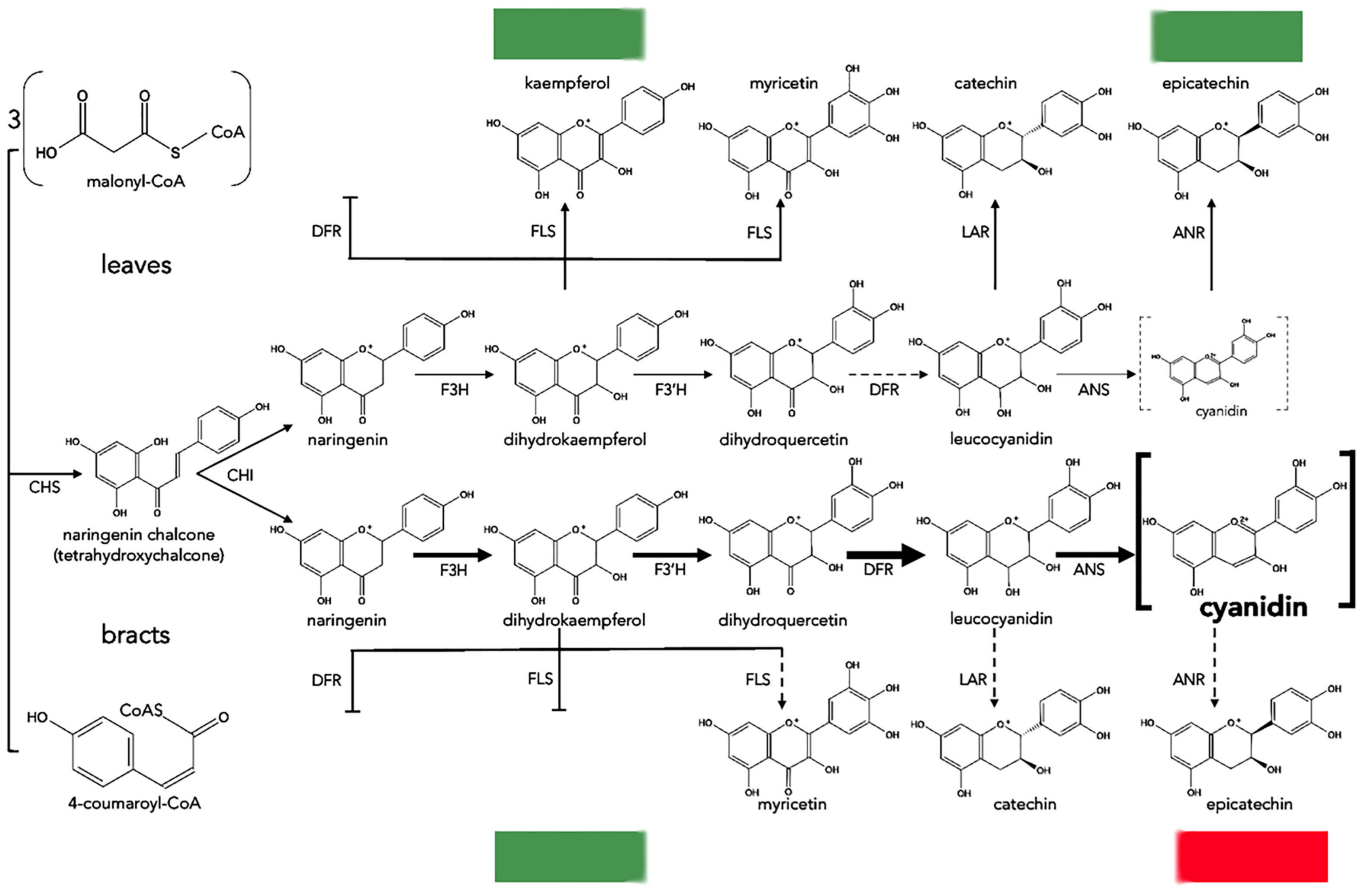
Proposed biosynthesis of anthocyanins in leaves and bracts of poinsettia. Dotted and blunt arrows indicate a decrease or absence of enzyme activity, respectively. The thickest arrows indicate preferential reactions ending with cyanidin. Dotted grey brackets mean a minimal amount of cyanidin, and bold brackets mean a high amount of cyanidin. CHS, chalcone synthase; CHI, chalcone isomerase; F3H, flavanone 3-hydroxylase; F3′H, flavonoid 3′-hydroxylase; DFR, dihydroflavonol 4-reductase; ANS, anthocyanidin synthase; ANR, anthocyanidin reductase; LAR, leucoanthocyanidin reductase; FLS, flavonol synthase.

The mechanism of the natural color change in poinsettias was recently investigated by Gu et al. (2018), who compared the content of metabolic products of the anthocyanin pathway and sequencing data obtained from the transcriptomes of green leaves and red bracts. Higher contents of flavonoids such as flavanones, flavones and flavonols were found in green leaves compared to red bracts, which predominantly contained more cyanidins and pelargonidins; this suggests that anthocyanin biosynthesis in green leaves is repressed. Transcriptome analysis identified 91,917 unique transcripts of which 72 were assigned to flavonoid biosynthesis, and among these it was observed that 3 unique transcripts of genes for key enzymes [CHS, CYP73A (trans-cinnamate 4-monooxygenase) and DFR], had higher expression levels in red bracts than in green leaves; this was also confirmed by real-time PCR (qPCR). In addition, the overexpression of poinsettia *DFR* increased the anthocyanin content in transformed *Arabidopsis thaliana* plants. Through a correlation analysis between the obtained results, *DFR* was identified as a promoter of anthocyanin accumulation in poinsettia (**Figure 4**).

## 10 Genetic modification of floral color: in search of attractive bluish varieties

The main driver of the floral industry is the development of new varieties; these provide market opportunities, increase productivity, and improve the quality of the final product (Chandler and Tanaka, 2007). In this sense, the color of the flower is one of the key traits for the consumer’s choice about the product and, although in nature they are exhibited in a wide range, most ornamental crops are restricted to a spectrum of color; in other words, they lack specific colors limited by their own genetics. Despite the conventional breeding by artificial crosses has been very successful, it still depends on laborious methods and requires long times. Moreover, in some species sterility occurs, preventing sexual reproduction and, in addition to this, certain important crops are out of reach for the development of new colors; for example, blue in flowers such as rose (*Rosa x hybrida*), carnation (*Dianthus caryophyllus*), chrysanthemum (*Chrysanthemum morifolium*), gerbera (*Gerbera* spp.) or poinsettia (Tanaka et al., 2008; Nishihara and Nakatsuka, 2010; Chandler and Brugliera, 2011; Zhao and Tao, 2015; Azadi et al., 2016). Therefore, there is a lot of interest in applying plant biotechnology techniques to ornamental products, not only to generate basic research, but also to manipulate the type of pigment expressed. To date, most research has focused on plant tissue culture methodologies and genetic modification to reach this goal (Davies and Schwinn, 2004; To and Wang, 2006).

The previously mentioned species do not have blue flowers due to the absence of the flavonoid 3’,5’hydroxylase (*F3’5’H*) gene in their genomes (Tanaka and Brugliera, 2014). In this sense, it is logic that the primary strategy to redirect anthocyanin biosynthesis towards delphinidin production is to introduce and induce the heterologous expression of the key gene *F3’5’H*, coming from various blue flowers. However, it is known that the accumulation of this pigment depends on the source of the transgene; for example, in tobacco the heterologous expression of the *F3’5’H* genes from *Campanula medium*, *Eustoma russellianum* or *Petunia x hybrida*, revealed that the *F3’5’H* gene from *C. medium* was more efficient for delphinidin production (Okinaka et al., 2003). Likewise, the *F3’5’H* gene of *Clitoria ternatea* was the best for delphinidin production in *Verbena hybrida* transgenic flowers, in comparison to the self *F3’5’H* gene of *V. hybrida* (± 30% and 10% of total anthocyanins, respectively) (Togami et al., 2006). In rose, the heterologous expression of the above five *F3’5’Hs* genes and those from *Gentiana* sp., *Cineraria* sp., or *Lavendula* sp., resulted in relatively similar delphinidin production ( ± 10%). Only roses containing the *Viola wittrockiana F3’5’H* transgene achieved a greater accumulation of delphinidin (± 60%) (Tanaka and Brugliera, 2013). Additionally, this last gene was introduced into chrysanthemums under the transcriptional control of different gene promoters such as those of *CHS* from *Gerbera hybrida*, *DFR*, *F3H* and *CHS* from *Rosa rugosa*, *F3’5’H* from *V. wittrickiana*, *F3H* from chrysanthemum and 35SCaMV, and resulted in a low delphinidin content ( ± 5%), except for flowers holding the *F3’5’H* transgene controlled by the chrysanthemum *F3H* promoter itself ( ± 30%) (Noda et al., 2013). These results suggest that is essential to choose the source of the transgene and the most appropriate promoter based on the biosynthesis of the new delphinidin anthocyanins in the plant of interest.

The production of delphinidin by heterologous expression of *F3’5’H* does not necessarily generate a significant phenotypic change in the color of the transgenic flower; some examples are in tobacco (Okinaka et al., 2003) or in lilium and petunia (Qi et al., 2013). The main reason for this failure is that these plants have other anthocyanins of the pelargonidin and/or cyanidin type, so the new F3’5’H enzyme must compete with other endogenous enzymes such as F3’H, FLS and DFR for the same DHK substrate. To overcome this competition, it has been proposed to use a *DFR* gene whose enzyme is preferential for dihydromyricetin (DHM), and/or to delete the genes of competing endogenous enzymes. A clear example is the reddish carnation, where isolated overexpression of the *PhF3’5’H* gene (*P. hybrida*), resulted in some delphinidin production, although it was not sufficient to change flower color. To eliminate competition between endogenous DFR and exogenous F3’5’H enzymes, a white (*DFR*-deficient) carnation was selected for transformation. Through the coexpression of the *PhF3’5’H* and *PhDFR* genes of *P. hybrida* (preferential reducer of DHM), under the control of the *CHS* promoter of *Antirrhinum* sp., it was possible to obtain pale purple flowers due to the exclusive accumulation of delphinidin (Holton, 1996). These Florigene^®^ Moondust™ carnations were the first transgenic ornamental crops commercialized in Japan and Australia (Tanaka et al., 1998); Subsequently, the Florigene^®^ Moonshadow™ line was developed, which showed a more intense purple color due to the co-expression of the *VwF3’5’H* gene from *V. wittrockiana* and *PhDFR* (Tanaka and Brugliera, 2013). Bluish color change was due to the co-pigmentation effect exerted between the new anthocyanin (delphinidin 3,5-diglucoside-6’’-*O*-4 and 6’’’-*O*1-cyclic malate) and flavone C-glucoside (apigenin 6-C-glucosyl-7-*O*-glucoside-6’’’-malate), and at relatively high vacuolar pH (Fukui et al., 2003). Ideal vacuolar conditions and an intermolecular interaction mechanism favored the establishment of bluish pigments.

The previous strategies served as the basis for further investigations; however, in certain plants such as roses or chrysanthemums, there are no white varieties especially devoid of DFR (Tanaka et al., 2009). In addition to this, reddish roses do not have flavones and the vacuolar pH is relatively low (Katsumoto et al., 2007). To resolve these unfavorable intracellular conditions, 169 rose cultivars were studied and those that showed higher vacuolar pH, low F3’H activity and higher accumulation of flavonols were chosen to carry out genetic transformation. Of the forty selected, six were transformed by expression of the *VwF3’5’H* gene under the CaMV35S promoter. Results showed that the percentage of delphinidin accumulation (32.4 - 82.3%) and the floral color change (magenta-lavender), depended on the intracellular conditions of the transformed cultivars. Subsequently, to optimize the heterologous expression of *F3’5’H*, two vectors were used: one containing the *VwF3’5’H* and *IhDFR* genes of *Iris hollandica*, and another that included the two previous ones plus the self-endogenous *RhDFR* gene of rose but used for RNA interference (RNAi) to cause silencing of *RhDFR*. Transgenic plants showed a more exclusive accumulation of delphinidin 3,5-diglucoside (98% of total anthocyanins), and a color change from pink to light purple when the latter polygenic vector was used (Katsumoto et al., 2007). These studies suggest that the selection of the most suitable cultivar for the establishment of the bluish color, the optimization of the transgenic expression through the incorporation of other genes that lead to the production of delphinidin and the suppression of competing endogenous genes are important factors to consider in the case of *E. pulcherrima* for anthocyanins metabolic engineering.

Although cultivars with new flower colors have been developed through these genetic manipulation strategies, the fact is that they still do not show a true-blue color. This reveals that, in addition to high delphinidin production, other factors such as the presence of co-pigments, delphinidin polyacylation, and a high vacuolar pH are perhaps necessary (Okitsu et al., 2018). This was clear in dahlia and orchid, where the simple isolated heterologous expression of the *F3’5’H* gene of *Commelina communis* produced floral varieties with colors between violet and blue (Nakano et al., 2016). These colors were achieved easily because the vacuoles of these plants were more appropriate for bluish pigments, since they contain a high pH and present polyacylated delphinidins with aromatic organic groups at the C-7,3’ positions, together with the presence of co-pigments that favor the stability and bathochromic shift of the pigment (Tanaka and Brugliera, 2014; Noda, 2018). To try to simulate the vacuolar conditions of blue flowers, the pH must be changed, or metal ions must be incorporated; however, introducing genes that regulate these complex processes is detrimental, and their function is still not fully understood (Tanaka and Brugliera, 2014). An alternative to generate a modified delphinidin, is by using genes encoding glycosyltransferase and/or acyltransferase enzymes, which add glucosyl-acyl residues to the anthocyanin A and/or B rings (Yoshida et al., 2009; Sasaki and Nakayama, 2015). Recently, in chrysanthemum (*Chrysanthemum morifolium*) (a cyanidin 3-[6’’-malonyl]-glucoside producer), the strategy was to generate a polyacylated delphinidin through the union of *p*-coumaroyl glucosyl residues, like ternatin D3 (delphinidin 3-[6’’-malonyl]-glucoside 3’,5’-di-*p*-coumaroyl glucoside), one of the anthocyanins of the Asian pigeonwings (*Clitoria ternatea*). For this, the genes *CmF3’5’H* of *C. medium* and *CtA3’,5’-GT* from *C. ternatea* were expressed with the *ChF3’H* promoter of chrysanthemum. The intention was to obtain a delphinidin 3-(6’’ -malonyl)-glucoside3’,5’-diglucoside, an anthocyanin called C5 that is precursor to D3. Unexpectedly, 19 of 32 (59%) of the transgenic flowers showed true blue coloration due to the production of delphinidin 3-(6’’-malonyl)-glucoside-3’,5’-diglucoside, while the rest were violet containing delphinidin 3-(6’’-malonyl)-glucoside. This indicates that the bluish coloration depends on the C-3’,5’ glycosylation of delphinidin. Given these results, the subsequent modifications of the anthocyanin through the addition of aromatic acyl groups were not necessary (Noda et al., 2017). Later, it was revealed that the mechanism for establishing the bluish pigment in the transgenic chrysanthemum was due to an intermolecular association established between delphinidin 3’,5’-diglucoside and flavone 7-glucoside derivatives such as luteolin and apigenin (co-pigmentation effect) (Noda, 2018). If the goal is to modify the bracts color from red to blue in *E. pulcherrima* all these experimental approaches should be considered.

## 11 Plant tissue culture of poinsettia

For the generation of stable transgenic plants of any plant species, it is essential to have efficient *in vitro* plant regeneration and plant genetic transformation protocols (Thieman and Palladino, 2010; Krenek et al., 2015). In poinsettia, various *in vitro* plant regeneration strategies have been established that include both regeneration pathways: organogenesis and embryogenesis (**Table 1**).

**Table 1:** Main investigations on *in vitro* culture of poinsettia (*E. pulcherrima*).

Regeneration	Variety	Explant	Use of plant growth regulators for the MG response	Results	Author
Indirect embryogenesis	‘Angelika’	Meristems	I. Callus induction: MS + BAP/CPA (0.2/0.2 mg L^-1^) II. Formation of somatic embryos: liquid MS + CPA/NAA/2iP (0.1/0.2/0.1 mg L^-1^) III. Embryo maturation: MS + BAP (0.05 mg L^-1^)	Regeneration of 3,000 complete plants with the use of bioreactors	Preil and Beck (1991); Geier et al. (1992); Grotkass et al. (1995)
Direct organogenesis	‘Freedom’ and ‘Lilio’	Apical meristems	I. Shoot proliferation: MS BAP/IAA (1.0/0.2 mg L^-1^) II. Root induction in basal MS	Obtaining shoots between 4-5 weeks of cultivation. 63% of this showed root formation	Bech and Rasmussen (1996)
Indirect embryogenesis	‘Angelika’	Hypocotyls	Induction of somatic embryos: MS + 2.0 mg L^-1^ of IAA	Generation of 1,400 embryos from 320 embryogenic calli. 8% developed normally	Osternack et al. (1999)
Indirect embryogenesis	Not reported	Nodes	I. Induction of callus and somatic embryos: MS + 2iP/NAA (2.0/0.5 mg L^-1^) II. Embryo maturation: 2iP/NAA (2.0/0.1 mg L^-1^)	Induction of embryogenic calluses at five weeks of culture, on average 0.2 per explant	Jasrai et al. (2003)
Direct and indirect organogenesis	‘Winter Rose’	I. Apical and lateral meristems II. Leaves	I. Shoot proliferation: MS + IAA/BAP (0.5/2.0 mg L^-1^) II. Callus and shoot induction: MS + BAP/IAA (2.0/3.0 mg L^-1^)	I. An average of 2.7 shoots per explant was reached II. 90% of explants with callus, from these 55% generated adventitious shoots	Pickens et al. (2005)
Indirect embryogenesis	‘Millenium’	Internode	I. Callus induction: MS + BAP/CPA (0.2/0.2 mg L^-1^) II. Embryo induction: MS + NAA/2iP (0.3/0.15 mgL^-1^) III. Embryo maturation: MS + BAP (0.05 mg L^-1^) IV. Root generation: MS 50% and sucrose 2%	I. Induction of 75% embryogenic calli from 350 inoculated explants II. Generation of 7.2 somatic embryos on average per callus III. Regeneration of 768 complete plants	Clarke et al. (2008)
Direct organogenesis	Various	Nodes	I. Shoot proliferation: MS + BAP/NAA (1.0/0.1 mg L-1) II. Root induction: MS + IAA (4.0 mg L^-1^)	I. Generation of 5 adventitious shoots on average per explant, with a 70% response II. Complete plants in a three-month period of cultivation	Castellanos et al. (2010)
Direct organogenesis	Not reported	I. Apical meristem II. Steams III. Leaves	I. Shoot proliferation: MS + BAP/adenine sulfate (0.5/20 mg L^-1^) II. Shoot induction: MS + BAP/NAA (1.0/0.2 mg L^-1^) III. Shoot induction: MS + BAP/IAA (1.0/0.2 mg L^-1^) IV. Root induction: MS + IAA (1.0 mg L^-1^)	I. Development of 10.6 shoots on average per explant II. Generation of 6.9 shoots on average per explant and 100% of explants generated shoots III. Induction of 3 shoots on average per explant and 77% of explants responded IV. 77% of shoots rooted	Danial and Ibrahim (2016)

MG (morphogenetic), MS (Murashige and Skoog, 1962), BAP (6-benzylaminopurine), CPA (4-chlorophenoxyacetic acid), NAA (1-naphthaleneacetic acid), 2iP (2-isopentenyladenine), IAA (indole-3-acetic acid).

The most studied route for the *in vitro* propagation of poinsettia is by direct organogenesis from the apical and axillary meristems. Shoot proliferation is commonly induced by adding a higher proportion of the cytokinin BAP (0.5-1.0 mg L^-1^) in relation to auxins such as IAA or NAA (0.1-0.2 mg L-1). The morphogenetic response of explants begins at the fourth week of cultivation and, once the shoot has developed, IAA (1.0-4.0 mg L^-1^) is supplemented to the culture medium to obtain a complete plant with roots; this process extends to the third month of cultivation. On average, between 3 and 5 shoots have been regenerated per inoculated explant (Bech and Rasmussen, 1996; Pickens et al., 2005; Castellanos et al., 2010). Interestingly, this average doubled (10.6 shoots per explant) with the use of BAP (0.5 mg L^-1^) and adenine sulfate (20 mg L^-1^) (Danial and Ibrahim, 2016). These authors also established a regeneration protocol by direct organogenesis from poinsettia explants, using a combination of BAP (1.0 mg L^-1^) and NAA (0.2 mg L^-1^). They achieved the induction of 6.9 and 3.0 adventitious shoots per stem and leaf explant, respectively. On the other hand, the indirect organogenesis route from leaf explants, was established by the induction of undifferentiated callus tissue and its subsequent redifferentiation. A higher concentration of IAA in relation to BAP was necessary to generate callus in 90% of the explants, and from these, 55% developed adventitious shoots (Pickens et al., 2005).

**Figure 5:**
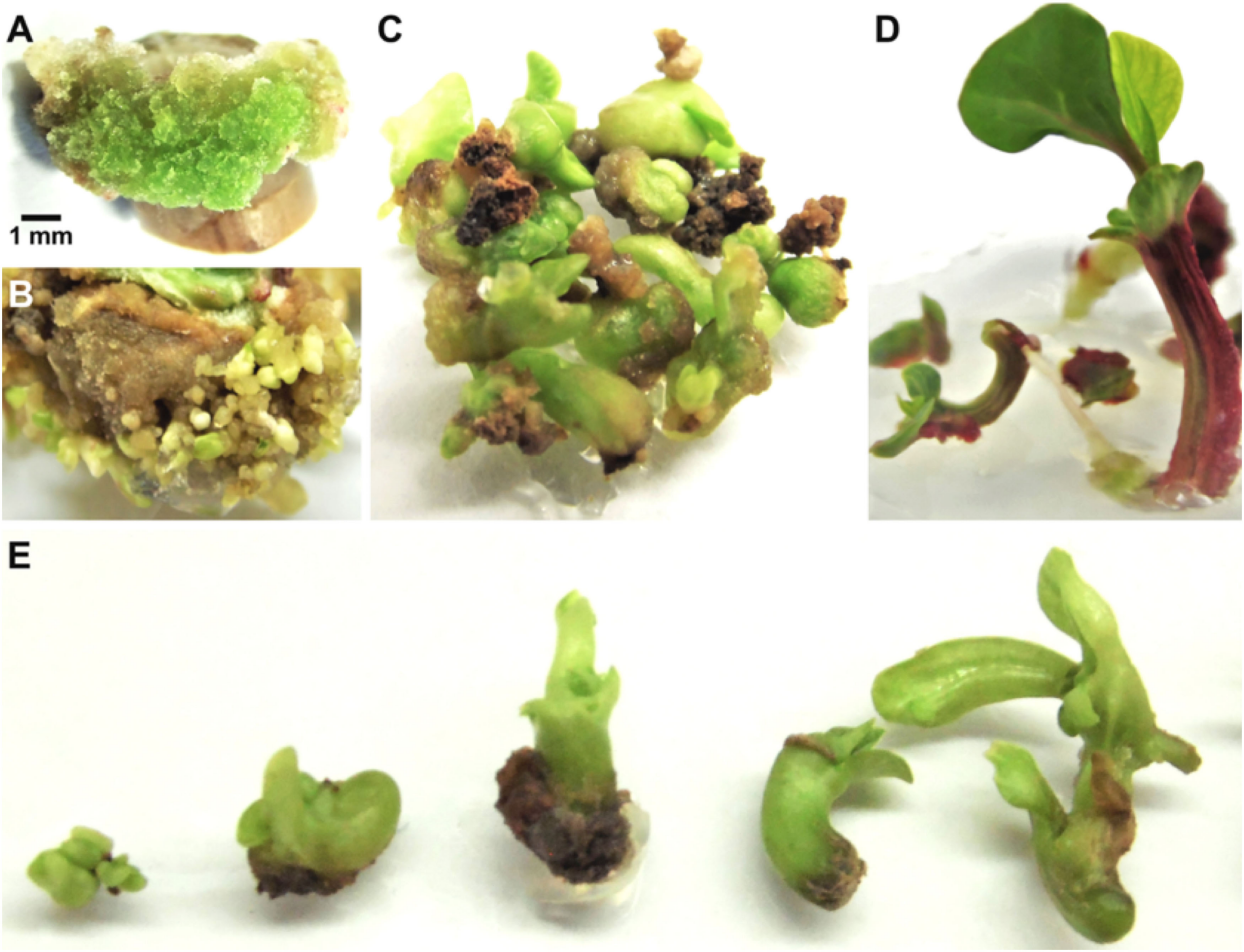
Somatic embryogenesis in poinsettia cv. Prestige Red. **(A)** Callus emerged from internode stem explants after 2 weeks of culture on MS medium supplemented with CPA/BAP (0.2/0.2 mg L^-1^). **(B)** Embryogenic callus formation after 13 weeks of culture on MS supplemented with NAA/2iP (0.3/0.15 mg L^-1^). **(C)** Proliferation of embryo structures at different developmental stages after 16 weeks of culture. **(D)** Growth of regenerated whole plants after 19 weeks of culture on MS supplemented with IAA (1.0 mg L^-1^). **(E)** Somatic embryos at different developmental stages.

Plant regeneration *via* the somatic embryogenesis pathway has been established from meristems (Preil and Beck, 1991; Geier et al., 1992; Grotkass et al., 1995; Jasrai et al., 2003), and from hypocotyl and internode tissues (Osternack et al., 1999; Clarke et al., 2008). In general, to induce embryogenic callus in poinsettia tissues it is common to combine BAP and the auxin *p*-chlorophenoxyacetic acid (4-CPA) at the same concentrations (0.2 mg L^-1^). For the induction of somatic embryos, the addition of cytokinin 2iP (0.1-0.15 mg L^-1^) has been used in combination with various auxins at different concentrations NAA, CPA (0.1-0.3 mg L^-1^) or IAA (2.0 mg L^-1^). Then, to mature these embryos of somatic origin, a cytokinin, commonly BAP (0.05 mg L^-1^), was used. This process was the best for the regeneration of 7.2 embryos per callus on average, where 75% of internode explants induced embryogenic callus within thirteen weeks of culture (Clarke et al., 2008). It should be addressed that morphogenetic capacity depends on the genetic background of any plant species, and in the case of *E. pulcherrima*, specific combinations of growth regulators must be tested to establish *in vitro* efficient plant regeneration protocols for those cultivars of particular interest. In **Figure 5** the *in vitro* plant regeneration process from node explants of *E. pulcherrima* cv. Prestige Red is shown.

## 12 Genetic transformation of *E. pulcherrima*

To date, the number of publications dealing with genetic transformation of poinsettia is very limited; a patent by Smith et al. (1997) reported the use of biolistics as a transformation method, in which it is described the isolation of apical and axillary meristems, *in vitro* inoculation, and embryogenic calluses induction, then these were bombarded with microparticles containing the expression vector. They induced somatic embryos on a selection medium and finally, mature embryos developed on two media to obtain transgenic poinsettias.

Vik et al. (2001) and Clarke et al. (2006) employed a transformation method based on *in vivo* electrophoresis, locating a pipette on a meristem which, in turn, was connected to a negative electrode. The pipette contained an agarose solution carrying an expression plasmid, which included the *GUS* reporter gene; the positive electrode was placed at the base of the stem, and a 0.5 mA current was set for 10 min. The results indicated that 9 of 34 evaluated shoots (26%), were GUS positive. However, a stable transgenic plant was never developed, regardless of the transiently elevated expression of the reporter gene (Clarke et al., 2008).

Islam et al. (2015) reported the genetic transformation of poinsettia mediated by *A. tumefaciens*. This was carried out by using freshly disinfected internodes explants, which were then inoculated and cocultivated with *A. tumefaciens* containing the expression plasmid. *E. pulcherrima* plants were then regenerated *via* embryogenesis, through the induction of embryogenic callus tissue, somatic embryos generation, maturation and rooting on selection medium with different hormonal balances. This stable genetic transformation methodology has been successfully used to develop transgenic poinsettias with resistance to *Poinsettia Mosaic Virus* (PnMV) using an RNA silencing strategy (Clarke et al., 2008), and compact-growth transgenic lines due to overexpression of the *A. thaliana AtSHI* (Arabidopsis short internode) gene (Islam et al., 2013) were also regenerated. These investigations reported a transformation frequency of 2.1% on average. Thus, this methodology of genetic transformation mediated by *A. tumefaciens* is proposed to achieve improvements in product quality, such as alteration of the color exhibited by the bracts, to satisfy consumer demands (Clarke et al., 2008; Islam et al., 2015).

## 13 A molecular approach for metabolic engineering of colors in poinsettia

Despite the high ornamental value of poinsettia, information on the molecular mechanisms underlying the establishment of bract color is relatively scarce; however, three research groups have reported some results. Gu et al. (2018) found a higher content of secondary flavonoids such as flavonoids, flavones and flavonols in the green leaf compared to the red bract, in which same flavonoids were found to decrease and the amounts of cyanidin and pelargonidin were increased. On the other hand, they showed that transcripts of three gene (*CHS*, *C4H* and *DFR*) had higher expression in the bract compared to the green leaf. These results indicate that promotion of DFR activity together with the inhibition of flavonoid secondary products, may result in the accumulation of anthocyanin pigments in the bracts (Gu et al., 2018). Based on these results, we suggest the use of the *DFR* promoter to generate constructs with foreign *F3’5’H* genes to transform and express them in *E*. *pulcherrima* specifically in the bracts under the short-day conditions as it occurs naturally.

On the other hand, to know the reason of the orange color in poinsettia, the anthocyanin content of the dark-red cultivars ‘Christmas Beauty’ (CB), ‘Christmas Feeling’ (CF), red-orange ‘Premium Red’ (PR) and ‘Harvest Orange’ (HO) was quantified. It was determined that the CB and CF cultivars contained a higher accumulation of cyanidin (69 and 72%, respectively); while the PR and HO cultivars showed a higher accumulation of pelargonidin (85 and 96%, respectively). Additionally, the *F3’H* and *DFR* cDNAs of each variety were isolated, corresponding recombinant enzymes were obtained, and their functional activity was studied. The kinetics showed that DFRs of the four varieties had higher substrate specificity for DHQ and DHM, compared to DHK. On the other hand, the cDNA of the *F3’H* genes encoded functional enzymes, except for the one from cv. HO, which had a 28 base pair insertion that causes a mutation due to a premature stop codon and, therefore, encodes an inactive enzyme. Together these results indicate that the red-orange varieties owe their color to a greater accumulation of pelargonidin and that, in cv. ‘HO’ this is due to an inactive F3’H enzyme (Nitarska et al., 2018). The mechanism of white coloration in poinsettia was reported by means of a comparative analysis of the transcriptomic sequences obtained from the ‘Christmas Feelings’ cultivar with red bracts, and the ‘Christmas Feelings Pearl’ cultivar with white bracts, in three stages of foliar development. The differential expression revealed that most of the structural anthocyanin biosynthesis-related genes were similarly expressed in both cultivars at the first stages of development. However, in the last stage, the *UGT79B10* (glycosyltransferase) and *GSTF11* (glutathione transferase) genes were less expressed in the white variety, a result validated by qRT-PCR analysis. This could explain the establishment of the white color in poinsettia (Vilperte et al., 2019). Finally, previous studies in our research group demonstrated that the purple fruits of the *Capsicum eximium* chili pepper exclusively accumulate delphinidin 3-coumaroylrutinoside-5-glucoside anthocyanins (Aza-González and Ochoa-Alejo, 2012). The coding sequences *CeF3’5’H* and *CeDFR* for these enzymes were isolated from 10 DPA (days post-anthesis) fruits, the developmental stage with the highest delphinidin content (149.7 μg g^-1^). The *CeF3’5’H-GUS* and *CeF3’5’H-CeDFR-GUS* constructs were generated for *A. tumefaciens*-mediated transformation in a transient and stable manner in tobacco and poinsettia (Cuéllar-González, 2019). The histochemical detection of GUS revealed a transient high expression in 100% of explants of poinsettia internode and tobacco leaves. In addition, 25 and 14 seedlings from embryogenic and organogenic origin, were *in vitro* regenerated on a selective medium (kanamycin), from 235 (10.6%) and 255 (5.5%) poinsettia explants, respectively. On the other hand, from 135 tobacco leaf segments, 98 (72.6%) of organogenic plants were regenerated on a selective medium. Using RT-PCR, the presence of *CeF3’5’H* and *CeDFR* transcripts were detected in 13 out of 20 (65%) evaluated tobacco plants; likewise, 13 of 19 (68%) revealed the presence of *CeF3’5’H*. Currently, all these putative transforming plants are in the process of characterization (Cuéllar-González, personal communication).

## 14 Genome editing of anthocyanin biosynthesis-related genes in diverse plant species and in *E. pulcherrima*

Genome editing is a powerful tool to specifically modify genes of interest or to study the specific gene function in different biological processes in plant species (Belhaj et al., 2015; Rojas-Vasquez and Gatica-Arias, 2020). Recently, this approach has been used to carry out genome editing of anthocyanin biosynthesis regulatory genes (transcription factors) (Cermák et al., 2015; Park et al., 2017; Wan et al., 2017; Xu et al., 2019; Yan et al., 2020; Yang et al., 2021) as well as of anthocyanin biosynthesis-related structural genes (Li et al., 2016; Watanabe et al., 2017; Danilo et al., 2018; Nishihara et al., 2018; Jung et al., 2019; Klimek-Chodacka et al., 2019; Tasaki et al., 2019) in different plant species mainly for gene function studies but also to generate changes in the color of some organs including flowers (**Table 2**). Regarding editing of anthocyanin biosynthesis-related genes specifically in *E. pulcherrima*, Nitarska et al. (2021) performed targeted mutagenesis of *F3’H* gene, necessary for cyanidin biosynthesis, in the red *E. pulcherrima* cv. ‘Christmas Eve’ using CRISPR/Cas9. As a result, they generated poinsettia plants with orange bract color due to the accumulation of pelargonidin instead of cyanidin. The *F3’H*-edited plants exhibited low cyanidin accumulation levels and a predominance of pelargonidin content giving a reddish orange color to the bracts.

**Table 2:** Investigations on CRISPR/Cas9 genome editing systems applied to color modification.

Organism	Gene target and function	Method	Results	Reference
*Solanum lycopersicum*	*Anthocyanin mutant 1* (*ANT1*) MYB transcriptional activator of anthocyanin biosynthesis	Knock-in	Tomatoes with *ANT1* overexpression, resulted in increased anthocyanin accumulation causing intense purple coloration in callus, flowers, fruits and foliage	Cermák et al. (2015)
*Arabidopsis thaliana*	*Production of Anthocyanin Pigments* (*PAP1*) MYB transcriptional activator of anthocyanin biosynthesis	Activation	Gene expression of *PAP1* was increased two- to three-fold and the activated plants showed purple pigmentation in the leaf	Park et al. (2017)
*Populus tomentosa*	*MYB57* MYB transcriptional repressor of anthocyanin biosynthesis	Knockout	*myb57* poplar mutants resulted in high anthocyanin and proanthocyanidin contents	Wan et al. (2017)
*Daucus carota*	*MYB113-like* MYB transcriptional activator of anthocyanin biosynthesis	Knockout	Edited purple carrots in the *MYB113-like* gene result in purple depigmented carrot plants. The *MYB113-like* affects the expression of gene involved in later steps of anthocyanin biosynthesis	Xu et al. (2019)
*Solanum lycopersicum*	*AN2-like* MYB transcriptional activator of anthocyanin biosynthesis	Knockout	Knockout of *AN2-like* gene led to a reduced content of anthocyanins in tomato fruits. This was associated with the downregulation of multiple anthocyanin biosynthesis-related genes	Yan et al. (2020)
*Solanum lycopersicum*	*MYBATV* Gene related to the repression of anthocyanin synthesis	Knockout	Knockout of *MYBATV* caused an increase in the anthocyanin content in tomato fruits, showing the fully purple phenotype. The anthocyanin biosynthesis-related genes were all highly expressed	Yan et al. (2020)
*Oryza sativa*	*Transparent Testa Glabra1* (*TTG1*) WD40 transcriptional activator of anthocyanin biosynthesis	Knockout	*ttg1* mutants of rice, exhibited a significantly decrease in anthocyanin accumulation in different organs (leaf blade, leaf sheath, collar, and root). In the grain only slight amount of anthocyanin was present	Yang et al. (2021)
*Arabidopsis thaliana*	Glycosyltransferases: *UGT79B2* and *UGT79B3* Structural genes	Knockout	Regenerated knockout lines *ugt79b2* and *ugt79b3* showed more susceptibility to stress and had lighter colors due to a minor anthocyanin level	Li et al. (2016)
*Ipomoea nil*	*DFR-B* Structural gene	Insertion and deletion	*Ipomea* mutants exhibited flower color change, from violet to white, due to the lack of anthocyanins caused by the lost in the activity of DFR enzyme	Watanabe et al. (2017)
*Solanum lycopersicum*	*DFR* Structural gene	Knockout and knock-in	Endogenous *DFR* gene was deleted by knockout, the callus-regenerated plants showed green instead of the typical purple color. Then, these tomato mutants were used to reinsert the *DFR* gene by knock-in, and they recovered their purple coloration	Danilo et al. (2018)
*Torenia fournieri*	*F3H* Structural gene	Knockout	*Torenia* plants mutated in the *F3H* gene generated flowers with notable visual color changes, from pale blue to white, different from blue wild type *Torenia*	Nishihara et al. (2018)
*Oryza sativa* L.	*F3’H*, *DFR* and Leucoanthocyanidin dioxygenase (*LDOX*) Structural genes	Knockout	CRISPR/Cas9 was applied to mutate three anthocyanin biosynthesis related genes. The phenotype of these mutants showed depigmented seeds and lower anthocyanin content	Jung et al. (2019)
*Daucus carota*	*F3H* Structural gene	Knockout	Carrot callus with mutated *F3H* was not capable of synthesizing anthocyanins and remained white, visually distinguished from purple wild-type cells	Klimek-Chodacka et al. (2019)
*Gentiana* x *hybrid*	Anthocyanin 5-glycosyltransferase (*A5-GT*), anthocyanin 3*’*-glycosyltransferase (*A3’-GT*) and anthocyanin 5,3*’*-aromatic acyltransferase (*A5,3’-AT*) Structural genes	Knockout	The flower color changed from vivid blue of wild-type plants to different color shade, due to predominance of different delphinidin derivates, like pale red violet, dull pink and pale mauve for the *A5-GT*, *A3′GT*, and *A5,3’AT* knockout lines, respectively	Tasaki et al. (2019)
*Gentiana* x *hybrid*	Glutathione S-transferase (*GST1*) Structural gene	Knockout	The *GST1* genome-edited lines exhibited two types of mutant flower phenotypes, almost white and pale blue. These phenotypes were associated with decreased anthocyanin accumulation in flower petals, especially the anthocyanidin glycoside gentiodelphin	Tasaki et al. (2020)
*Euphorbia pulcherrima*	*F3’H* Structural gene	Knockout	*F3’H* gene-edited poinsettia plants exhibited a bract color change from red to vivid reddish orange. There was due to a higher accumulation of pelargonidin-based anthocyanins instead of cyanidin	Nitarska et al. (2021)
*Petunia* x *hybrid*	*F3H* Structural gene	Knockout	Doble knockout plants *f3ha f3hb* exhibited a clearly visible modified flower color from vivid purple to pale purple pinkish	Yu et al. (2021)

## 15 Conclusion

*Euphorbia pulcherrima* is a very important commercial ornamental plant, which has been domesticated and improved through traditional breeding techniques to generate cultivars with inflorescences bearing bracts exhibiting red, orange, pink or white colors due to the accumulation or not of anthocyanins of the cyanidin or pelargonidin type. However, since there are established *in vitro* plant regeneration and genetic transformation protocols for *E. pulcherrima*, it is possible to manipulate the anthocyanin biosynthesis pathway to produce poinsettia plants with bluish bracts by using biotechnological tools and foreign anthocyanin biosynthesis-related genes under the control of endogenous gene promoters to express them properly.

## 16 Perspectives

*Euphorbia pulcherrima* is a worldwide cultivated ornamental plant species, which has been subjected to different genetic breeding programs through traditional methods to generate cultivars with desired growth habits, forms, and bract colors. These characteristics have been modified through very time-consuming improvement programs. However, it is possible to generate modern poinsettia cultivars by using biotechnological tools such as tissue culture combined with genetic transformation or genome editing approaches to short the time to do it. In the case of metabolic engineering of anthocyanin biosynthesis in poinsettia to render plants with different bract colors, a special attention should be paid to the efficiency improvement of the current reported *in vitro* plant protocols as wells as those for genetic transformation because they are cultivar dependent and are usually long time-consuming techniques. It should be also convenient to investigate and characterize the structural genes and their respective enzymes to understand the biochemistry and molecular biology of this pathway. Until now, very limited information has been reported on this (Gu et al., 2018; Nitarska et al., 2018), and even less has been investigated regarding the transcriptional regulation of this pathway in poinsettia. It is well known in some other plant species that the anthocyanin biosynthesis pathway is regulated by a complex formed by MYB-MYC (bHLH)-WD40 transcription factors (Petroni and Tonelli, 2011; Falcone Ferreyra et al., 2012); whether it is the same mechanism or there are some variations in poinsettia is still unknown. By knowing the biochemical and molecular biology basis of the anthocyanin’s biosynthesis pathway in poinsettia it will allow, in a better way, the metabolic engineering of this process in poinsettia; for example, the overexpression of some structural or transcription factor genes could lead to an over-accumulation of some final specific products (anthocyanins), whereas the stable gene silencing or the genome editing of some key genes should produce plants with different bracts color as an interesting trait for commercial purposes. Furthermore, basic studies on genes involved in the anthocyanin biosynthetic pathway in poinsettia might reveal the best promoters to be used for the gene constructs, which should be expressed in the proper tissue (bracts) and at the most adequate time (flowering); this could be the case of the *DFR* gene promoter instead of using a constitutive promoter such as the 35S derived from the *Cauliflower Mosaic Virus*. All these possibilities to genetically manipulate *E. pulcherrima* to produce plants with bluish-bracts should certainly face the acceptance or reluctance from the potential consumers.

## Author contributions

Conceptualization, EL-G and NO-A. Methodology and investigation, FC-G. Resources, EL-G and NO-A. Writing: original draft, EL-G, NO-A, and FC-G. Writing: review and editing, EL-G and NO-A. Supervision, EL-G and NO-A. All authors have read and agreed to the published version of the manuscript.
